# Coastal acidification impacts on shell mineral structure of bivalve mollusks

**DOI:** 10.1002/ece3.4416

**Published:** 2018-08-14

**Authors:** Susan C. Fitzer, Sergio Torres Gabarda, Luke Daly, Brian Hughes, Michael Dove, Wayne O'Connor, Jaimie Potts, Peter Scanes, Maria Byrne

**Affiliations:** ^1^ Institute of Aquaculture University of Stirling Stirling UK; ^2^ School of Medical Sciences University of Sydney Sydney New South Wales Australia; ^3^ School of Geographical and Earth Sciences University of Glasgow Glasgow UK; ^4^ Hunter Local Land Services Taree New South Wales Australia; ^5^ New South Wales Department of Primary Industries Fisheries NSW Port Stephens Fisheries Institute Taylors Beach New South Wales Australia; ^6^ Estuaries and Catchments Science NSW Office of Environment and Heritage Sydney South New South Wales Australia; ^7^ School of Life and Environmental Sciences University of Sydney Sydney New South Wales Australia

**Keywords:** acidification, biomineralization, bivalve mollusks, oysters, shell structure, sulfate soil

## Abstract

Ocean acidification is occurring globally through increasing CO_2_ absorption into the oceans creating particular concern for calcifying species. In addition to ocean acidification, near shore marine habitats are exposed to the deleterious effects of runoff from acid sulfate soils which also decreases environmental pH. This coastal acidification is being exacerbated by climate change‐driven sea‐level rise and catchment‐driven flooding. In response to reduction in habitat pH by ocean and coastal acidification, mollusks are predicted to produce thinner shells of lower structural integrity and reduced mechanical properties threatening mollusk aquaculture. Here, we present the first study to examine oyster biomineralization under acid sulfate soil acidification in a region where growth of commercial bivalve species has declined in recent decades. Examination of the crystallography of the shells of the Sydney rock oyster, *Saccostrea glomerata*, by electron back scatter diffraction analyses revealed that the signal of environmental acidification is evident in the structure of the biomineral. *Saccostrea glomerata*, shows phenotypic plasticity, as evident in the disruption of crystallographic control over biomineralization in populations living in coastal acidification sites. Our results indicate that reduced sizes of these oysters for commercial sale may be due to the limited capacity of oysters to biomineralize under acidification conditions. As the impact of this catchment source acidification will continue to be exacerbated by climate change with likely effects on coastal aquaculture in many places across the globe, management strategies will be required to maintain the sustainable culture of these key resources.

## 1 INTRODUCTION

Ocean acidification as a result of climate change threatens the process of mollusk shell biomineralization through the reduction of carbonate available for shell production and by challenging metabolic processes and energetic partitioning (Doney, Fabry, Feely, & Kleypas, 2009; Gazeau et al., 2007). Calcifying organisms, such as shellfish, are at most risk from ocean acidification, as carbonate is vital in the biomineralization of their calcium carbonate protective shells (Gazeau et al., 2007). The upwelling of high CO_2_ water and reduction in aragonite saturation state (aragΩ) along the United States Pacific Northwest was responsible for hatchery failures and mass mortality of oyster larva and has deleterious effects on growth of shellfish (Barton et al., 2015; Ekstrom et al., 2015). This upwelling of high CO_2_ water, which is a natural feature of the local oceanographic system, is being exacerbated by climate change driven increased in pCO_2_ levels. Experimental acidification studies widely report reduced shell growth in mussels and oysters, including reduced shell thickness and mechanically weaker shells (Beniash, Ivanina, Lieb, Kurochkin, & Sokolova, 2010; Dickinson et al., 2012; Fitzer et al., 2015; Gazeau et al., 2007; Ries, 2011; Ries, Cohen, & Mccorkle, 2009). These changes in the shell present concerns for shellfish growth and defense against predators. It is thought that acidification impacts shell growth and mechanical properties through a shift to a disorganized crystallographic structure (Beniash et al., 2010; Dickinson et al., 2012; Fitzer et al., 2015; Hahn et al., 2012) reducing the structural integrity and the ability of shellfish to biomineralize (Fitzer et al., 2015). These findings have been seen in experimental ocean acidification experiments through scanning electron imaging (SEM), microhardness testing and calcification rates (Beniash et al., 2010; Dickinson et al., 2012; Fitzer et al., 2015; Gazeau et al., 2007; Ries et al., 2009) and transplantation of bivalves to natural CO_2_ vents (Hahn et al., 2012).

In addition to ocean acidification, many near shore marine habitats are also exposed to freshwater runoff with lowered pH due to leachate from acid sulfate soils and humic/tannic acids from groundwaters. The extent of this coastal acidification is being exacerbated by climate change‐driven sea‐level rise and catchment‐driven flooding and land runoff. Decaying plant matter leaches tannic and humic acids, which are mildly acidic organic polyphenols (Jiang et al., 2017). Acid sulfate soils have a much greater potential for large pH reductions due to iron pyrite (FeS_2_) which, when excavated or drained, oxidizes on exposure to oxygen and generates sulfuric acid (Dent, 1986; Dent & Pons, 1995). The chemistry mechanisms of sulfate soil acidification and ocean acidification are very different (Box 1). In sulfate soil acidification, sulfuric acid is produced through oxidation reactions (Dent & Pons, 1995; Box 1), which alters environmental total alkalinity. In an important way, the oxidation of Fe^2 +^ can occur at some distance from the original source of pyrite in drainage and floodwaters which can further decrease estuarine pH (Dent & Pons, 1995). In contrast, CO_2_‐induced acidification occurring from freshwater input and atmospheric CO_2_ absorption causes carbonic acid acidification which alters environmental dissolved inorganic carbon (Doney et al., 2009). Considering the dynamic nature of coastal habitats, Duarte et al. (2013) question whether the relatively small changes in oceanic pH due to climate change are relevant against a background of large aquatic pH changes due to photosynthesis, catchment runoff, and other drivers.

Box 1 **Equations for mechanisms of sulfate soil and CO_2_ induced acidification**

1 Mechanisms of sulfate soil‐induced acidification:(1)${\text{FeS}}_{2(s)}+7/2\;O_{2(\text{aq})}+H_{2}O\to {\text{Fe}}^{2+}{}_{\left(\text{aq}\right)}+2{\text{SO}}_{4}{}^{2-}{}_{\left(\text{aq}\right)}+2H^{+}{}_{\left(\text{aq}\right)}$
(2)${\text{Fe}}^{2+}\left(\text{aq}\right)+1/4\;O_{2(\text{aq})}+3/2\;H_{2}O\to \text{FeO}\cdot {\text{OH}}_{\left(s\right)}+2H^{+}{}_{\left(\text{aq}\right)}$

Mechanisms of CO_2_‐induced acidification:${\text{CO}}_{2}+H_{2}O↔H_{2}{\text{CO}}_{3}↔{\text{HCO}}_{3}{}^{-}+H^{+}↔{\text{CO}}_{3}{}^{2-}+2H^{+}$

Soil sourced sulfuric acid can be mobilized into nearby waterways during wet periods and cause estuarine acidification (Sammut, White, & Melville, 1996). Tidal seawater inundation, likely to increase with global sea level rise, has been linked experimentally to severe acidification through leaching and mobilization of trace elements in soils (Keene, Johnston, Bush, Burton, & Sullivan, 2010; Wong et al., 2010). This is a global problem often associated with the soil attributes of wetlands and mangrove forests (Hossain & Nuruddin, 2016) and is reported to cause damage to fisheries and shellfish culture in Sierra Leone, South America, Malaysia, Vietnam, Indonesia, and Australia (Blume, 1983; Dent & Pons, 1995; Klepper, Chairuddin, & Iriansyah, 1992; Michael, 2013; O'Connor & Dove, 2009; Sonnenholzner & Boyd, 2000).

Mollusk aquaculture is a $19 billion Global industry that produces 16.1 million tonnes annually (FAO, 2016). Oysters and mussels comprise the bulk of mollusk aquaculture and are considered vulnerable to climate change‐related acidification as this limits biomineralization for shell growth with the potential that this will be exacerbated by acidification from acid sulfate soils (Dove & Sammut, 2007a, 2007b). We investigated the impact of this form of coastal acidification on oyster biomineralization in the Sydney rock oyster, *Saccostrea glomerata*, in a region where the growth of this species has been in decline over recent decades. The region is prone to coastal acidification driven by sulfate soils in wetlands and the adjacent coastal floodplain that cause extremely low surface water pH and can release a large amount of postflood CO_2_ into waters (Jeffrey, Maher, Santos, Mcmahon, & Tait, 2016; Perkins, Santos, Sadat‐Noori, Gatland, & Maher, 2015; Webb et al., 2016). This estuarine acidification has the potential to impact oyster shell growth through reduced salinity, pH, and total alkalinity (Supporting Information Table S1; Dove & Sammut, 2007b; Smith & Heggie, 2003). Over recent years, there has been a decrease in oyster production and shell size, leading to a decrease in production of larger, higher value, “plate” grade oysters and an increase in the smaller “bistro” and “bottle” grade oysters (O'Connor & Dove, 2009).

We characterized the crystallography of the shells of *S. glomerata* resident in habitats with a range of pH using high‐resolution electron back scatter diffraction (EBSD) to assess oyster shell growth through changes in the calcite crystal biomineralization (Fitzer, Cusack, Phoenix, & Kamenos, 2014; Fitzer, Phoenix, Cusack, & Kamenos, 2014). In what appears to be the first study of shell crystallography for the oyster species, we use this approach to determine if there is a signal of environmental acidification caused by sulfate soil outflows in the structure of the biomineral, as there is in mussels grown under CO_2_‐driven acidification (Fitzer, Cusack, et al., 2014; Fitzer, Phoenix, et al., 2014). We investigated oysters from commercial populations of oyster family lines developed for aquaculture (O'Connor & Dove, 2009) grown under different levels of estuarine acidification. Our findings highlight the potential disruption of crystallographic control over biomineralization in Sydney rock oysters grown in Australian coastal acidification sites.

## 2 MATERIALS AND METHODS

**Figure 1:**
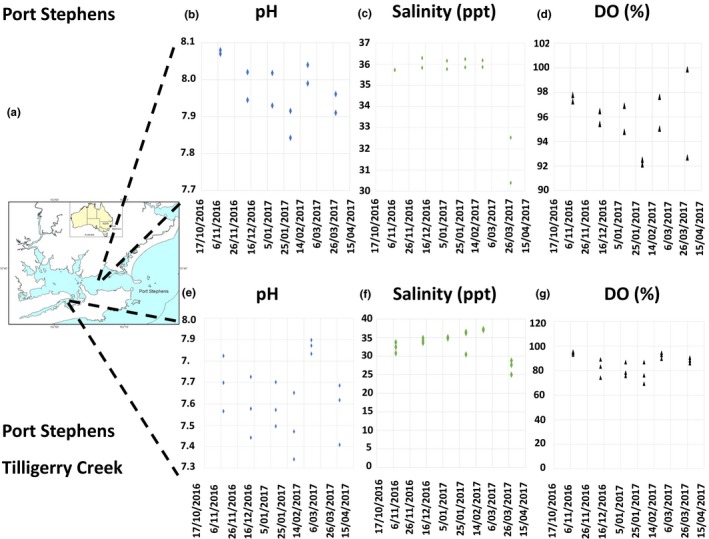
(a) Map of Port Stephens Estuary showing the Port Stephens control and Tilligerry Creek acidified sites. The mean values of pH, salinity (ppt), and dissolved oxygen (%) are shown for each site (control b–d) acidified (e–g). The impact of runoff from a rainfall event on pH and salinity can be seen in the data for 29/03/17 [Colour figure can be viewed at http://wileyonlinelibrary.com]

**Figure 2:**
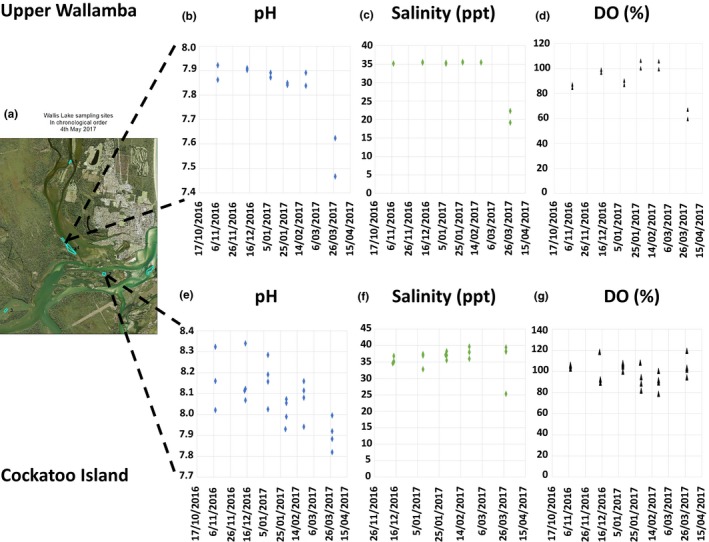
(a) Map of Wallis lake Estuary showing the Wallis Lake control and acidified sites. The mean values of pH, salinity (ppt), and dissolved oxygen (%) are shown for each site (control b–d) (acidified e–g). The impact of runoff from a rainfall event on pH and salinity can be seen in the data for 29/03/17 [Colour figure can be viewed at http://wileyonlinelibrary.com]

We investigated oysters in two subtropical estuary systems that are major oyster farming areas, Wallis Lake and Port Stephens located in the mid‐north coast of New South Wales (NSW; Figure 2). Wallis Lake is responsible for 30% of Sydney rock oyster production (Livingston, 2017). The Wallamba River, which flows into the estuary, has 69% of its catchment cleared for agriculture (NSW National Parks and Wildlife Service, 2013). Port Stephens (Figure 1), is the second largest Sydney rock oyster production area responsible for a further 15% of industry output (Livingston, 2017). Tilligerry Creek, Port Stephens is a low‐lying floodplain containing a drainage network which discharges into Port Stephens estuary through a catchment area (130 km^2^) containing disturbed acid sulfate soils detrimental to Sydney rock oyster production (Port Stephens Council, 2008). Representative measurements of estuarine acidification are presented in the Supporting Informations for NSW Port Stephens and Wallis Lake sites. The Port Stephens oyster growing areas have been sampled extensively for water quality pH profiles to quantify the extent and duration of estuarine acidification (Dove & Sammut, 2013). The Port Stephens low pH site has a median pH of 7.28, at 22.86°C (range pH 4.31–8.44) (Dove & Sammut, 2013).

Water quality data were collected in Port Stephens, Tilligerry Creek, and Wallis River as part of routine estuary health monitoring by NSW Office of Environment and Heritage (OEH; Hallett et al., 2016; OEH, 2016). These data (Figures 1 and 2, Supporting Information Tables S1 and S2) facilitate understanding of water chemistry mechanisms operating at the study sites. Salinity, pH, dissolved oxygen (DO), and fDOM (fluorometric dissolved organic matter) data were measured with a calibrated EXO‐2 sonde in water from 0.5 m depth. At 2–3 “zones” within each of the sites, the EXO sonde measured each variable at 1 s intervals continuously for 5 min as the boat drifted with the wind. The sampling zone therefore consisted of a random direction transect 100–200 m long through the zone. A mean and standard deviation for each variable at the zone was calculated for each time. Data were collected six times (approx. 3 weeks apart) across the austral summer (November 2016–March 2017) (Supporting Information Table S2).

### 2.1 Oyster sampling

Sydney rock oysters were sampled from three commercial oyster leases in Wallis Lake and Port Stephens, New South Wales, Australia in the first week of May 2017. Port Stephens was sampled with a tidal height of 0.60–0.70 meters (Min tidal height 0.25 m, max 1.75 m), and at Wallis Lake, the tidal height was 0.50 meters (Min 0.4 m to max 1.50 m). The sites were chosen to reflect different exposures to acidification, but all had oysters grown from the same oyster family (F31). Sites in Wallis Lake were a “control” site “Cockatoo Island,” with estuarine salinities and good oyster growth; and the Upper Wallamba “acidified site 1,” a site which receives runoff from identified areas of acid sulfate soil in the Wallamba river and has poor oyster growth. In Port Stephens, the coastal acidified site was Tilligerry Creek, “acidified site 2,” also a site which receives runoff from acid sulfate soils. The oysters collected from each site (*n* = 8) were 2–3 years old and of similar size (Mean = 54.2 mm shell length, *SE* = 6.28 mm, *n* = 12). Three oysters were sampled per replicate site, 3 were used for SEM analyses and 3 were used for isotope analyses.

### 2.2 Oyster shell preparation for scanning electron microscopy‐electron backscatter diffraction

Oysters were dissected, shells rinsed with freshwater and air dried. The left “cupped” valve was embedded in epoxy resin, sliced longitudinally with a diamond trim slow saw and polished for scanning electron microscopy. In brief, the exposed cut shell section was polished using grit papers (P320, P800, P1200, P2500, and P4000), using polishing cloths with alpha alumina 1 μm and alpha alumina 0.3 μm, and finally using colloidal silica for 1 hr using a Vibromat. Shells were finished with distilled water and methanol. Polished shell sections were analyzed using SEM combined with EBSD. The same midsection of the shell from the outer calcitic layer to the inner chalky layers was selected for each individual. Specimens were examined under low vacuum mode (∼50 Pa) with an accelerating voltage of 20 kV using a Carl Zeiss Sigma variable pressure field emission gun SEM at the School of Geographical and Earth sciences, University of Glasgow, UK. The stage was tilted to 70° to examine backscatter Kikuchi patterns (Perez‐Huerta & Cusack, 2009). Energy dispersive X‐ray spectroscopy (EDS) was used to investigate sulfur incorporation into the shells and representative SEM‐EDS sulfur maps were constructed. Simultaneous energy dispersive X‐ray spectroscopy (EDS) and EBSD data were collected by an Oxford Instruments X‐Max 80 mm^2^ Silicon Drift Detector and a NordlysMax^2^ EBSD detector respectively over an area ~1 mm^2^/sample with a step size of 1 μm. EBS patterns were analyzed in Oxford Instruments Aztec 3.3 software using a 4 × 4 binning and an exposure time of ~70 ms and a frame average of 1 frame to facilitate rapid data acquisition. The mean angular deviation (MAD), an assessment of the quality of the pattern indexing where <1 is considered good, was 0.78 (Average MAD across 7 datasets, with a range of 0.74–0.84) for calcite. The data were noise reduced using Oxford Instruments HKL software Channel 5 by a wildspike correction that removes isolated datapoints followed by a 6‐point nearest neighbor zero solution. This procedure aids in defining grains without creating substantial artifacts. Crystallographic preferred orientations for calcite grains are defined by the two major crystallographic axis (with the following standard notation): {0001} and {$10\overset{¯}{1}0$}. Subsets of each data set were created for texturally distinct regions within the oyster shells. The poles to the major crystallographic planes in these subsets were plotted in a lower hemisphere stereographic projection using the mambo module in Oxford Instruments HKL software Channel 5.

### 2.3 Crystallographic misorientation

The crystallography of the shells grown under control and acidified conditions was compared using the m‐index calculated from the normalized abundance of uncorrelated misorientation angles (Supporting Information Figure S1). The m‐index was calculated for each specimen following the equations in (Skemer, Katayama, Jiang, & Karato, 2005) from 10,000 uncorrelated misorientations where a value of 1 relates to a perfect single crystal and 0 relates to a random misorientation of crystals. Misorientations were adapted from those determined for the hexagonal crystal system (Grimmer, 1979).

## 3 RESULTS

### 3.1 Wallis Lake and Port Stephens water chemistry

The Port Stephens Lake control site had a mean pH 7.98 (*SE* = 0.07, range pH 7.91–8.08, *n* = 12) from November 2016 to March 2017, while the acidified site 2 near Tilligerry Creek had a mean pH 7.63 (*SE* = 0.16, range pH 7.34–7.90, *n* = 18) (Figure 1, Supporting Information Table S2). Salinity at the control sites in Port Stephens had a mean of 35.21 ppt (*SE* = 1.82, range salinity 30.40–36.29 ppt, *n* = 12) and for Tilligerry Creek a mean of 33.40 ppt (*SE* = 3.54, range salinity 25.04–37.32 ppt, *n* = 18). At the acidified site there was a correlation between declining pH and decreased salinity (Figure 1, Supporting Information Table S2). The DO in the Port Stephens “acidified site 2” is also much lower with a mean of 85.79% saturation (*SE* = 7.88%, range of DO 69.41%–95.37%, *n* = 18) compared to the average DO for Port Stephens lake of 95.71% saturation (*SE* = 2.40%, range of DO 92.12%–97.78%, *n* = 12).

In Wallis Lake, the Upper Wallamba is representative of the sampling area for “acidified site 1” and the Cockatoo Island representative of the “control” site. pH in the “acidified site 1” had a mean of pH 7.82 (*SE* = 0.14, range of pH 7.47–7.92, *n* = 12) compared to a mean pH of 8.08 (*SE* = 0.13, range of pH 7.82–8.34, *n* = 23) in the “control” site. The salinity also followed a similar trend declining alongside the pH at the “acidified site 1” with a mean of 32.95 ppt (*SE* = 5.73, range of salinity 19.20–35.54 ppt, *n* = 12) compared to the “control” site with a mean of 35.40 (*SE* = 3.77, range of salinity 25.33–39.38 ppt, *n* = 23). The DO again was much lower in the “acidified site 1” with a mean of 90.29% saturation (*SE* = 14.54, range of DO 59.63%–106.31%, *n* = 12) compared to the “control” site with a mean DO of 99.19% saturation (*SE* = 10.40, range of DO 81.96–119.68%, *n* = 23) in Wallis Lake (Supporting Information Table S2, Figure S2).

The BC image is a visual representation of the quality of the received electron backscatter patterns (EBSP) where brighter areas produced better EBSP and shows the crystal structure from the ornamented calcite on the outer shell (bottom of the left image). The “feather like” calcite prisms more commonly imaged using EBSD, the inner most chalk layers (Top of Figure 2) in the oyster are also highlighted brighter with higher porosity.

### 3.2 Shell crystallography

**Figure 3:**
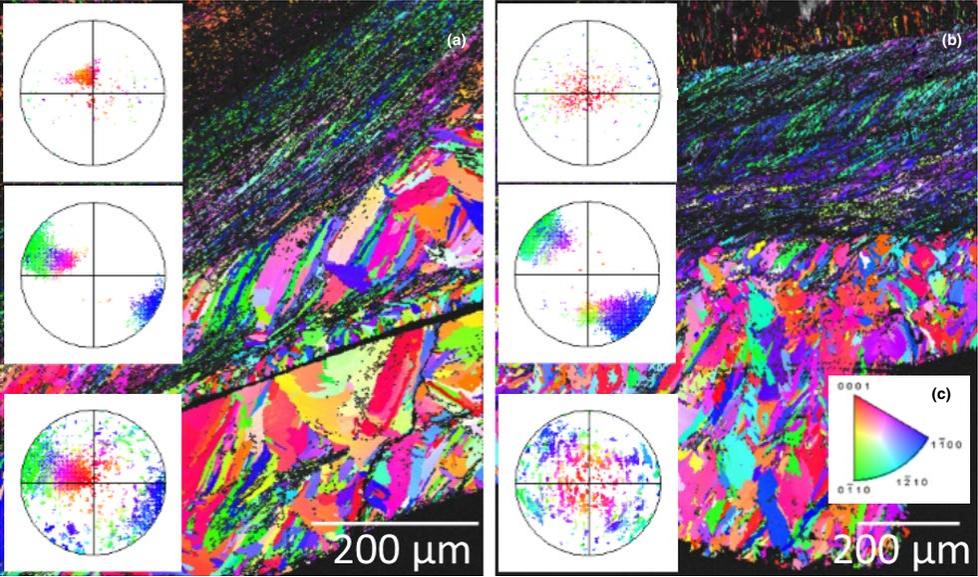
Inverse pole figure maps and associated pole figures of the Sydney rock oysters (specimen 1, a, and specimen 2, b) collected from the Wallis Lake “control” site Cockatoo Island. The colors indicate the crystallographic orientation relative to the plane of the section as per the color key for calcite (c). The pole figures are stereographic projections of the poles to the {0001} crystallographic plane of the calcite and correspond to the overlaid section of the color map [Colour figure can be viewed at http://wileyonlinelibrary.com]

**Figure 4:**
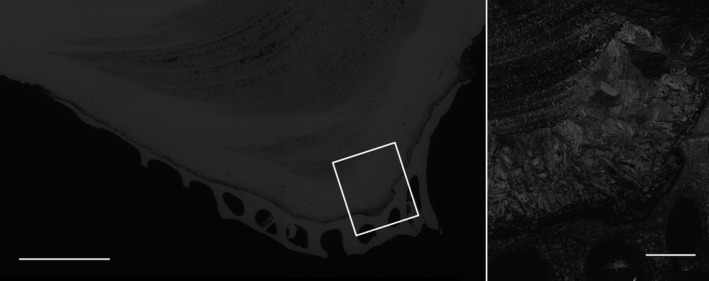
Backscattered electron image (left, scale 1 mm) and band contrast image (right, scale 200 μm) of the Sydney rock oyster (specimen 1) collected from the Wallis Lake “acidified site 1”(Upper Wallamba) from Figure 3a. The white box shows the region analyzed by electron backscatter diffraction

The shells of the Sydney rock oysters from the “control” site at Cockatoo Island are comprised of calcite prisms and chalk that produced good quality EBSP (MAD < 1) during EBSD data acquisition (Figure 3). The crystal layering of the calcite within the oysters is a complex structure; the outer most prismatic layers can be seen at the bottom of Figure 3. Moving toward the inner growth of the shell the layers become increasingly ordered and finer grained with interspersed layers of chalk (Figure 3). An example of this structure can be seen in Figure 4 as imaged using scanning electron microscopy, prior to the EBSD analysis of the shell section in Figure 3a. This is the first reporting of the crystallographic structure of calcite within the Sydney rock oyster shell. The chalky and fine‐grained calcite layers toward the growth of the shell are strongly aligned as evidenced by the point maxima in the poles to the {0001} plane (Figure 3). The {0001} plane of the fine‐grained calcite is perpendicular to the growth direction of the oyster while {0001} plane of the chalk contains the growth vector (Figure 3). The coarse prismatic calcite in the outer layer exhibits a slightly weaker alignment of the {0001} plane (Figure 3a) or a planar fabric as evidenced by the split girdle maxima in the poles to the {0001} plane (Figure 3b).

**Figure 5:**
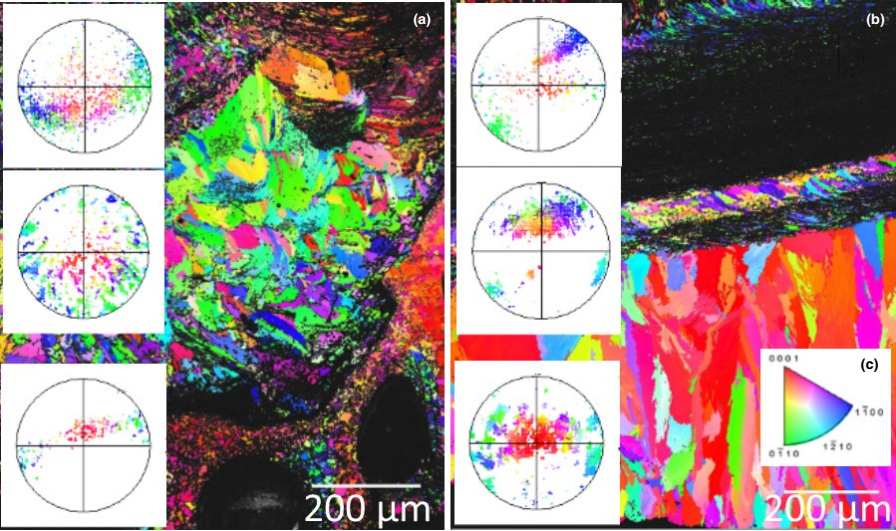
Inverse pole figure maps and associated pole figures of the Sydney rock oysters (specimen 1, a, and specimen 2, b) collected from the Wallis Lake “acidified site 1” (Upper Wallamba). The colors indicate the crystallographic orientation relative to the plane of the section as per the color key for calcite (c). The pole figures are stereographic projections of the poles to the {0001} crystallographic plane of the calcite and correspond to the overlaid section of the color map [Colour figure can be viewed at http://wileyonlinelibrary.com]

In comparison the shells of the Sydney rock oysters grown under coastal acidification in Wallis Lake at “acidified site 1” appear disordered throughout the outer prismatic layer (Figure 5a middle pole figure, and Figure 5b, bottom pole figure and the inner calcite folia, middle pole figure), while the chalky layers here have a planar fabric as evidenced by the girdle maxima (Figure 5, top pole figure). There is no weak alignment of the {0001} plane perpendicular to the growth direction as was observed in the middle folia of the oysters grown under “control” conditions in Cockatoo Island, Wallis lake (Figure 1).

**Figure 6:**
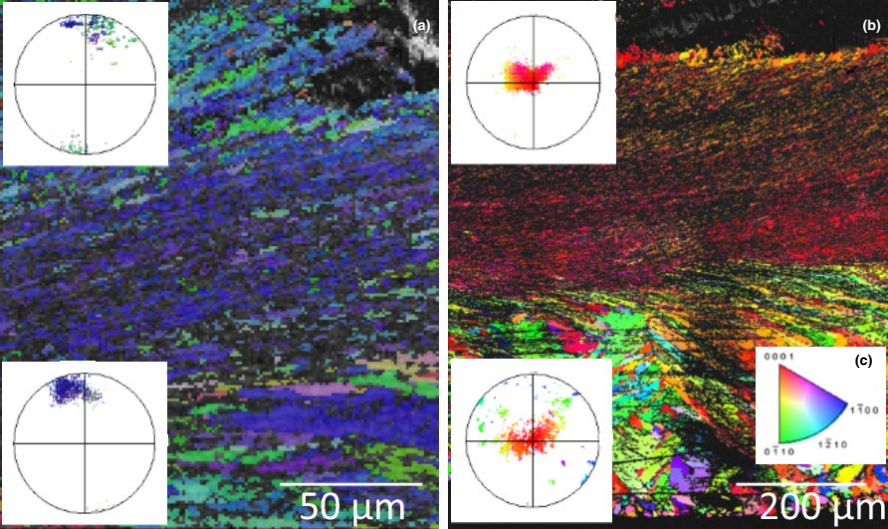
Inverse pole figure maps and associated of the Sydney rock oysters (specimen 1, a, and specimen 2, b) collected from the Port Stephens estuary “acidified site 2” (Tilligerry Creek). The colors indicate the crystallographic orientation relative to the plane of the section as per the color key for calcite (c). The pole figures are stereographic projections of the poles to the {0001} crystallographic plane of the calcite and correspond to the overlaid section of the color map [Colour figure can be viewed at http://wileyonlinelibrary.com]

The shells of the Sydney rock oysters grown at “acidified site 2” in Port Stephens show similarities in the chalky layer crystallographic growth compared to oysters grown in “control” conditions at Cockatoo Island (Figures 3 and 6). The crystallographic disorder seen in oysters grown under the acidification in Wallis lake “acidified site 1” is not as prominent in those grown under less acute acidification at Port Stephens “acidified site 2.” The same crystal growth patterns were seen to be consistent in several individuals (Figure 6a and b, *n* = 3).

#### 3.2.1 Crystallographic misorientation

The m‐index values for each of the specimens illustrated in Figures 3, 5, and 6 provide evidence for a more random misorientation of crystallography in the oysters from the Wallis Lake “acidified site 1” compared to those from the “control” Cockatoo Island site (Table 1). The m‐index values show that there can be large variability among individuals (e.g., Tilligery Acidified site 2’ Specimen 1).

**Table 1:** M‐index calculated for each specimen from *n* = 10,000 uncorrelated misorientations per individual oyster where a value of 1 relates to a perfect single crystal and 0 relates to a random misorientation of crystals

Specimen	m‐index
“Control” specimen 1	0.098
“Control” specimen 2	0.108
“Acidified site 1” Specimen 1	0.08
“Acidified site 1” Specimen 2	0.083
“Acidified site 2” Specimen 1	0.392
“Acidified site 2” Specimen 2	0.097

#### 3.2.2 Sulfur analysis by energy dispersive X‐ray spectroscopy

All the oysters examined had a similar levels of sulfur (<0.2 wt%) in their shells as shown by the peak in the mass spectrum, albeit at the detection limit of the SEM‐EDS. There was no visual increase in sulfur levels in oyster shells grown at acidified sites 1 or 2, Representative SEM‐EDS sulfur maps of the shells are provided in the Supporting Informations (Supporting Information Figure S1).

## 4 DISCUSSION

Coastal acidification is a widespread concern for coastal aquaculture (Dent & Pons, 1995; Dove & Sammut, 2007b; O'Connor & Dove, 2009; Sonnenholzner & Boyd, 2000) yet there are few studies on the impacts of this form of decreased pH on the growth of the shells and skeletons of cultured species and almost no consideration of how this stressor will interact with climate change impacts. Here we show that a commercially important oyster, the Sydney rock oyster, *S. glomerata*, shows phenotypic plasticity in response to coastal acidification driven by acid sulfate soil outflows. Growing under coastal acidification conditions causes a disruption of crystallographic control over biomineralization. The response of the biomineralization system seen here is similar to that shown by mussels grown under CO_2_‐driven acidification (Fitzer, Cusack, et al., 2014; Fitzer, Phoenix, et al., 2014; Hahn et al., 2012). Reduced growth of *Saccostrea glomerata* at the very low pH sites (pH 7.4) can be explained by a disruption to the biomineralization process, reducing the ability of the oyster to control the crystallographic layering of the calcite folia and chalky layers.

This is the first study to examine oyster shell crystallography and how it changes under acid sulfate soil induced acidification. The crystallography of the prismatic layers of *S. glomerata* from control (pH 8.21) sites does not resemble that reported for the Pacific oyster *Magallana gigas* (previously *Crassostrea gigas*) also grown in good estuarine conditions (Macdonald, Freer, & Cusack, 2010). For this latter species, the calcite prisms only exhibited a weak alignment of the {0001} plane perpendicular to the growth direction, similar to the fine‐grained calcite layers here (Macdonald et al., 2010). The crystallography presented for calcite layers in *M. gigas* show a single orientation in the calcite {0001} plane and crystallography remained uniform throughout the shell for oysters grown in good estuarine conditions (Macdonald et al., 2010) dissimilar to the crystallography of the *S. glomerata* presented in this study.

As found here for *S. glomerata* a similarly disorganized crystallographic shell structure has been observed by EBSD in mussels *Mytilus edulis* grown under experimentally elevated CO_2_ acidification (pH 7.2–7.7) (Fitzer, Cusack, et al., 2014; Fitzer, Phoenix, et al., 2014; Fitzer et al., 2015). The levels of crystallographic misorientation were variable among *S. glomerata* individuals from the acidified sites. To determine trends the shells of more individuals would need to be examined. In oysters, *Crassostrea virginica* changes to growth have been observed using scanning electron microscopy and microhardness testing (Beniash et al., 2010; Dickinson et al., 2012) grown under experimentally elevated CO_2_ acidification (pH = 7.5–7.9) these are at similar pH levels to the coastal acidification in this study. Experimental acidification studies report a reduction in the structural integrity of mussel and oyster shells leading to reduced mechanical properties of the shells (Beniash et al., 2010; Dickinson et al., 2012; Fitzer et al., 2015). This has the potential to increase vulnerability to predation and protection from mechanical forces such as storms. It is thought that the reduced structural integrity and shell growth may be due to metabolic demand as part of the physiological responses to the effects of acidification on acid‐base balance and biomineralization (Beniash et al., 2010; Fitzer, Cusack, et al., 2014; Fitzer et al., 2015; Gobler & Talmage, 2014).

It has been suggested in many experimental acidification studies that physiological impacts on metabolism increasing energy demand would leave less energy available for growth resulting in smaller oyster sizes (Beniash et al., 2010; Gobler & Talmage, 2014). The reduced oyster shell growth at sites in this study could be attributable to the limited capacity of oysters to biomineralize under the acid sulfate soil‐driven acidification conditions, shown through observations of crystallography for the first time in the Sydney rock oyster. In addition, oyster feeding rates are inhibited by acid sulfate soil outflows (Dove & Sammut, 2007a). Where we have seen reduced growth in Wallis Lake more smaller “bistro” and “bottle” grade oysters have been produced for commercial sale (O'Connor & Dove, 2009). There are a number of factors limiting the growth of oysters in NSW including disease (QX disease, parasite—*Marteilia sydneyi*), declines in water quality (including acid sulfate soils) and changes to single‐seed oyster production (Dove & Sammut, 2007b; O'Connor & Dove, 2009). There is no evidence of disease contributing to the decrease in oyster production at our study sites (Dove & Sammut, 2007b; Nell & Perkins, 2006). Acidified waters through acid sulfate soil floodplains are likely the dominant factor affecting mechanisms of oyster shell growth.

Ocean acidification induced growth effects on oyster shell biomineralization is reported for the Eastern oyster *C. virginica* using microhardness testing and scanning electron imaging (Beniash et al., 2010; Dickinson et al., 2012; Gobler & Talmage, 2014). In this study, we present similarly induced growth effects on shell biomineralization in the Sydney Rock oyster using an alternative crystallographic approach to understand mechanisms of oyster growth. Climate change‐driven acidification is likely to affect oyster shell biomineralization on a global scale and here we show that this may be exacerbated by coastal acidification from acid soil drainage.

This study shows that commercial oysters grown under both ocean acidification experiments and coastal acidification show similarly induced shell growth changes. This is the first study to examine the mechanisms behind this reduced growth in commercial oysters observing the disruption of crystallographic control over biomineralization using EBSD. Although similar growth responses occur in the oysters grown under these two types of acidification, it should be noted that the seawater chemistry mechanisms behind CO_2_ induced ocean acidification and sulfate soil induced coastal acidification are very different. As such, one would expect mechanisms behind the shell growth to differ between the two different forms of acidification. To understand the mechanistic response of crystallographic growth further research would require comparison of the same species grown in sulfate soil acidification and CO_2_ induced ocean acidification conditions. Sulfate soil acidification is caused by sulfuric acid produced through oxidation reactions (Dent & Pons, 1995). Although we expected to see sulfur incorporation into the shell biomineral, the SEM‐EDS investigation indicated very low sulfur levels and that these did not differ between oysters from control and acidified sites. In the bivalve *Artica islandica* sulfur bands were observed subannually (Shirai et al., 2014) similar to the sulfur map produced in shells grown at Tilligerry Creek, associated with organic content. In brachiopods, sulfur has been shown to be indicative of the sulfated organic matrix (England, Cusack, & Lee, 2007) and in the giant clam sulfur in aragonitic shells was suggested as the result of cyclic changes of shell growth (Yoshimura et al., 2013). However, as for the clam study (Shirai et al., 2014), there was no clear difference in sulfur concentration between growth bands for the Sydney rock oysters.

Acidification of coastal waters is driven by a number sources including—CO_2_ influenced ocean acidification, acid sulfate soil runoff, and runoff of humic/tannic acids. Diurnal pH fluctuations, which can exceed 1 pH unit, are often driven by the daily respiration and photosynthesis cycle of aquatic algae and macrophytes (Duarte et al., 2013). Thus, to distinguish the acidification signal from significant background changes, the acidification signal would need to be large. Climate change CO_2_ driven changes in pH of the order of 0.1 pH units have already occurred in world's oceans and a reduction of a further 0.3–0.5 units (pH 7.8–7.6) is expected by 2100 (Scanes, Parker, O'Connor, Stapp, & Ross, 2017). Humic/tannic acid changes in NSW are commonly around the 0.5–1 pH unit range (OEH unpublished data). In the 1950s, large drains were constructed to mitigate floods which altered the biodiversity and hydrology of the wetland complex (Creighton, 2013). Historically, the water in the wetland had a pH of 2 but due to remediation efforts, the pH as measured by Sammut et al. (1996) is around pH 6. The acidification (mean pH 7.63, range 7.34–7.90), and the consequent observed changes to shell structure and oyster growth, is well outside the range expected due to climate change alone and is therefore most likely influenced by drainage of acidified waters through nearby acid sulfate soil floodplains as well as runoff of humic/tannic acids.

The water quality data offer some insight as to the possible sources. The observed decreasing pH with decreasing salinity can be driven by enhanced organic matter remineralization in the water column, humic acids from ground water and inputs of water from acid sulfate soils. The reduced DO in the “acidified site 1” and “acidified site 2” (compared to controls) could be indicative of oxidation reactions caused by sulfate soil acidification or be due to organic matter remineralization in the water column. The fDOM data (Supporting Information Table S2) shows that there is a distinct increase in dissolved organic matter fluorescence when salinity decreases. This indicates the input of tannin water with associated tannic/humic acids is due to freshwater runoff. The carbonate chemistry at “acidified site 1” and “acidified site 2” differs from the control, indicated by a reduced total alkalinity, reduced carbonate, aragonite and calcite saturation, and an increase in pCO_2_ (Supporting Information Table S1). This could be influenced by freshwater input producing more DIC.

Based on the above data and the absolute magnitudes of pH change, we conclude that the lowered pH at our sample sites is due to a mixture of acid sulfate soil drainage and humic/tannic acid associated with freshwater runoff. The acid sulfate soil drainage is most likely a continuous chronic input and humic/tannic acids an additional pulse following rainfall.

It is important to note that future climate change has the potential to exacerbate both oceanic (CO_2_) acidification and coastal (acid sulfate soil and humic/tannic) acidification. Coastal acidification could increase as sea‐level rise results in tidal pumping of groundwater from greater areas of acid sulfate soil and increased runoff due to altered storm intensity (MEMA, 2017).

We have highlighted the problems of acid sulfate soil‐driven acidification for oyster fisheries, a phenomenon that causes problems for marine resources in many coastal systems with reduced fish growth and reduced abundances of fish in the Philippines (Blume, 1983; Klepper et al., 1992) and reduced shrimp growth and survival in Ecuador (Sonnenholzner & Boyd, 2000). There are relatively few studies focusing on the impact of sulfate soil acidification on shellfish aquaculture outside of Australia with the Sydney rock oyster (Amaral, Cabral, & Bishop, 2012; Dove & Sammut, 2007a, 2007b; O'Connor & Dove, 2009). The impact of estuarine acidification on the reduced growth of fish in fisheries has been the predominant focus in developing countries in South Asia (Blume, 1983; Dent & Pons, 1995; Klepper et al., 1992). Shrimp and bivalve shellfish aquaculture is becoming a promising, fast‐growing animal food‐producing sector to contribute to global food security and economic growth (FAO, 2012, 2016). The impacts of sulfate soil estuarine acidification on mechanisms of oyster growth have been shown in this study to be an issue which needs highlighting. However, maybe more importantly, it is vital to use information from commercial shellfisheries to plan for sustainable growth under climate change‐induced acidification. It is uncertain how the combined effects of ocean and coastal acidification will impact marine resources as the marine environment continues or be altered by climate change. In the historic case of the NSW oyster growers, industry moved to selling smaller oysters, and the reasons behind this move are unclear (O'Connor & Dove, 2009). It is not clear whether this reduced growth is a linear trend based on a slower rate of growth in acidified environments or whether the new mechanism of shell growth may be less energetically costly to maintain shell growth. Further research would be required targeting a cohort of oysters deployed at sites with different levels of acidification. Therefore, perhaps early harvesting of smaller “bistro” and “bottle” sized oysters could be a way forward for globally sustaining oyster growth and this may be applicable to other locations. However, selling small oysters has a direct impact on the profitability of oyster farms.

Climate change‐driven acidification has potential to affect oyster shell biomineralization and structural properties on a global scale as shown for *Crassostrea virginica* (Beniash et al., 2010; Dickinson et al., 2012), and here, we show that this may be exacerbated by coastal acidification from acid soil drainage. It is vital to consider and compare the mechanisms behind reduced shell growth in the differently induced acidification environments, oceanic and coastal, to predict future implications for commercial oyster aquaculture (Ellis, Urbina, & Wilson, 2017). If both ocean acidification and coastal acidification are exacerbated by future climate change and sea‐level rise, then perhaps this could have additive ramifications for commercial shellfish aquaculture.

## CONFLICT OF INTEREST

None declared.

## AUTHOR CONTRIBUTIONS

Susan Fitzer (SCF) and Maria Byrne (MB) designed and undertook field ecosystem experiments. SCF, MB, Sergio Torres Gabarda (STG), Brian Hughes (BH), and Michael Dove (MD) were all involved in the acquisition of field data. SF and Luke Daly (LD) analyzed field samples and interpreted the microscopy data. SF interpreted the data and drafted the manuscript with MS. Jaimie Potts (JP) and Peter Scanes (PS) collected field water quality data for the revised manuscript. SF, MS, BH, MD, JP, PS and Wayne O'Connor (WO) revised the work critically for important intellectual content and approved the final version to be published.

## DATA ACCESSIBILITY

Crystallographic orientation data from inverse pole figure maps are archived at the Dryad Digital Repository: https://doi.org/10.5061/dryad.cn006gh.

## References

[ece34416-bib-0001] Amaral, V. , Cabral, H. N. , & Bishop, M. J. (2012). Moderate acidification affects growth but not survival of 6‐month‐old oysters. Aquatic Ecology, 46, 119–127. 10.1007/s10452-011-9385-5

[ece34416-bib-0002] Barton, A. , Waldbusser, G. G. , Feely, R. A. , Weisberg, S. , Newton, J. , Hales, B. , … McLauglin, K. (2015). Impacts of coastal acidification on the Pacific Northwest shellfish industry and adaptive strategies implemented in response. Oceanography, 28, 146–159. 10.5670/oceanog

[ece34416-bib-0003] Beniash, E. , Ivanina, A. , Lieb, N. S. , Kurochkin, I. , & Sokolova, I. M. (2010). Elevated level of carbon dioxide affects metabolism and shell formation in oysters *Crassostrea virginica* . Marine Ecology Progress Series, 419, 95–108. 10.3354/meps08841

[ece34416-bib-0004] Blume, H. P. (1983). H. Dost and N. van Breemen (Eds.): Proceedings of the Bangkok symposium on acid sulphate soils. ILRI publications 31, Wageningen, Niederlande, 1982, 450 Seiten. Zeitschrift für Pflanzenernährung und Bodenkunde, 146, 130 10.1002/(ISSN)1522-2624

[ece34416-bib-0005] Creighton, C. (2013). Revitalising Australia's Estuaries. In: *FRDC 2013 Fisheries Research and Development Corporation* pp. 1–165, Retrieved from https://www.fishhabitatnetwork.com.au/userfiles/Revitalising%20Aus%20estuaries%20-%20smaller.pdf, Fisheries Research and Development Corporation

[ece34416-bib-0006] Dent, D. (1986). Acid sulphate soils: A baseline for research and development. Wageningen, the Netherlands: International Institute for Land Reclamation and Improvement/ILRI.

[ece34416-bib-0007] Dent, D. L. , & Pons, L. J. (1995). A world perspective on acid sulphate soils. Geoderma, 67, 263–276. 10.1016/0016-7061(95)00013-E

[ece34416-bib-0008] Dickinson, G. H. , Ivanina, A. V. , Matoo, O. B. , Portner, H. O. , Lannig, G. , Bock, C. , … Sokolova, I. M. (2012). Interactive effects of salinity and elevated CO_2_ levels on juvenile eastern oysters, *Crassostrea virginica* . The Journal of Experimental Biology, 215, 29–43. 10.1242/jeb.061481 22162851

[ece34416-bib-0009] Doney, S. C. , Fabry, V. J. , Feely, R. A. , & Kleypas, J. A. (2009). Ocean acidification: The other CO_2_ problem. Annual Review of Marine Science, 1, 169–192. 10.1146/annurev.marine.010908.163834 21141034

[ece34416-bib-0010] Dove, M. , & Sammut, J. (2007a). Histologic and feeding response of Sydney rock oysters, Saccostrea glomerata, to acid sulfate soil outflows. Journal of Shellfish Research, 26, 509–518. 10.2983/0730-8000(2007)26%5b509:HAFROS%5d2.0.CO;2

[ece34416-bib-0011] Dove, M. C. , & Sammut, J. (2007b). Impacts of estuarine acidification on survival and growth of Sydney rock oysters, *Saccostrea glomerata* (GOULD 1850). Journal of Shellfish Research, 26, 519–527. 10.2983/0730-8000(2007)26%5b519:IOEAOS%5d2.0.CO;2

[ece34416-bib-0012] Dove, M. C. , & Sammut, J. (2013). Acid sulfate soil induced acidification of estuarine areas used for the production of Sydney rock oysters, *Saccostrea glomerata* . Journal of Water Resource and Protection, 5, 16.

[ece34416-bib-0013] Duarte, C. M. , Hendriks, I. E. , Moore, T. S. , Olsen, Y. S. , Steckbauer, A. , Ramajo, L. , … McCulloch, M. (2013). Is ocean acidification an open‐ocean syndrome? Understanding anthropogenic impacts on seawater pH. Estuaries and Coasts, 36, 221–236. 10.1007/s12237-013-9594-3

[ece34416-bib-0014] Ekstrom, J. A. , Suatoni, L. , Cooley, S. R. , Pendleton, L. H. , Waldbusser, G. G. , Cinner, J. E. , … Portela, R. (2015). Vulnerability and adaptation of US shellfisheries to ocean acidification. Nature Clim. Change, 5, 207–214. 10.1038/nclimate2508

[ece34416-bib-0015] Ellis, R. P. , Urbina, M. A. , & Wilson, R. W. (2017). Lessons from two high CO2 worlds – future oceans and intensive aquaculture. Global Change Biology, 23, 2141–2148. 10.1111/gcb.13515 27762490 PMC5434897

[ece34416-bib-0016] England, J. , Cusack, M. , & Lee, M. R. (2007). Magnesium and sulphur in the calcitic shells of two brachiopods, *Terebratulina retusa* and *Novocrania anomala* . Lethaia, 40, 2–10.

[ece34416-bib-0017] FAO (2012). Food and Agriculture Organization of the United Nations Annual report. Fishery and Aquaculture Statistics. pp.65.

[ece34416-bib-0018] FAO (2016). The State of World Fisheries and Aquaculture 2016. In: *Contributing to food security and nutrition for all* pp.1–190, Rome.

[ece34416-bib-0019] Fitzer, S. C. , Cusack, M. , Phoenix, V. R. , & Kamenos, N. A. (2014). Ocean acidification reduces the crystallographic control in juvenile mussel shells. Journal of structural biology, 188, 39–45. 10.1016/j.jsb.2014.08.007 25180664

[ece34416-bib-0020] Fitzer, S. C. , Phoenix, V. R. , Cusack, M. , & Kamenos, N. A. (2014). Ocean acidification impacts mussel control on biomineralisation. Scientific Reports, 4, 6218. 25163895 10.1038/srep06218 PMC5385834

[ece34416-bib-0021] Fitzer, S. C. , Zhu, W. , Tanner, K. E. , Kamenos, N. A. , Phoenix, V. R. , & Cusack, M. (2015). Ocean acidification alters the material properties of *Mytilus edulis* shells. Journal of the Royal Society Interface, 12, 20141227. 10.1098/rsif.2014.1227 PMC4305426 25540244

[ece34416-bib-0022] Gazeau, F. , Quiblier, C. , Jansen, J. M. , Gattuso, J.‐P. , Middelburg, J. J. , & Heip, C. H. R. (2007). Impact of elevated CO_2_ on shellfish calcification. Geophysical Research Letters, 34, 1–5.

[ece34416-bib-0023] Gobler, C. J. , & Talmage, S. C. (2014). Physiological response and resilience of early life‐stage Eastern oysters (Crassostrea virginica) to past, present and future ocean acidification. Conservation Physiology, 2, cou004 10.1093/conphys/cou004 27293625 PMC4732497

[ece34416-bib-0024] Grimmer, H. (1979). The distribution of disorientation angles if all relative orientations of neighbouring grains are equally probable. Scripta Metallurgica, 13, 161–164. 10.1016/0036-9748(79)90058-9

[ece34416-bib-0025] Hahn, S. , Rodolfo‐Metalpa, R. , Griesshaber, E. , Schmahl, W. W. , Buhl, D. , Hall‐Spencer, J. M. , … Immenhauser, A. (2012). Marine bivalve shell geochemistry and ultrastructure from modern low pH environments: Environmental effect versus experimental bias. Biogeosciences, 9, 1897–1914. 10.5194/bg-9-1897-2012

[ece34416-bib-0026] Hallett, C. S. , Valesini, F. J. , Scanes, P. , Crawford, C. , Gillanders, B. M. , Pope, A. , … Maxwell, P. (2016). A review of Australian approaches for monitoring, assessing and reporting estuarine condition: II. State and Territory programs. Environmental Science and Policy, 66, 270–281. 10.1016/j.envsci.2016.07.013

[ece34416-bib-0027] Hossain, M. D. , & Nuruddin, A. A. (2016). Soil and mangrove: A review. Journal of Environmental Science and Technology, 9, 198–207. 10.3923/jest.2016.198.207

[ece34416-bib-0028] Jeffrey, L. C. , Maher, D. T. , Santos, I. R. , Mcmahon, A. , & Tait, D. R. (2016). Groundwater, acid and carbon dioxide dynamics along a coastal wetland, lake and estuary continuum. Estuaries and Coasts, 39, 1325–1344. 10.1007/s12237-016-0099-8

[ece34416-bib-0029] Jiang, T. , Kaal, J. , Liang, J. , Zhang, Y. , Wei, S. , Wang, D. , & Green, N. W. (2017). Composition of dissolved organic matter (DOM) from periodically submerged soils in the Three Gorges Reservoir areas as determined by elemental and optical analysis, infrared spectroscopy, pyrolysis‐GC–MS and thermally assisted hydrolysis and methylation. Science of The Total Environment, 603–604, 461–471. 10.1016/j.scitotenv.2017.06.114 28641186

[ece34416-bib-0030] Keene, A. F. , Johnston, S. G. , Bush, R. T. , Burton, E. D. , & Sullivan, L. A. (2010). Reactive trace element enrichment in a highly modified, tidally inundated acid sulfate soil wetland: East Trinity, Australia. Marine Pollution Bulletin, 60, 620–626. 10.1016/j.marpolbul.2010.02.006 20223484

[ece34416-bib-0031] Klepper, O. , Chairuddin, G. T. , & Iriansyah, Rijksen H. D. (1992). Water quality and the distribution of some fishes in an area of acid sulphate soils, Kalimantan, Indonesia. Hydrobiological Bulletin, 25, 217–224. 10.1007/BF02270806

[ece34416-bib-0032] Livingston, S. (2017). Aquaculture Production Report 2015–2016. pp.1–11. ISSN 1444‐840.

[ece34416-bib-0033] Macdonald, J. , Freer, A. , & Cusack, M. (2010). Attachment of oysters to natural substrata by biologically induced marine carbonate cement. Marine Biology, 157, 2087–2095. 10.1007/s00227-010-1476-7

[ece34416-bib-0034] MEMA (2017). NSW Marine Estate Threat and Risk Assessment – Background Environmental information. pp.409. ISBN 978‐1‐74256‐983‐3.

[ece34416-bib-0035] Michael, P. S. (2013). Ecological impacts and management of acid sulphate soil: A review. Asian Journal of Water, Environment and Pollution, 10, 13–24.

[ece34416-bib-0036] Nell, J. A. , & Perkins, B. (2006). Evaluation of the progeny of third‐generation Sydney rock oyster Saccostrea glomerata (Gould, 1850) breeding lines for resistance to QX disease Marteilia sydneyi and winter mortality Bonamia roughleyi. Aquaculture Research, 37, 693–700. 10.1111/j.1365-2109.2006.01482.x

[ece34416-bib-0037] Nsw National Parks and Wildlife Service (2013). Wallis lake nature reserves draft plan of management. (ed Heritage OOEA ), pp. 1–37. ISBN 978 1 74359 253 3.

[ece34416-bib-0038] O'Connor, W. , & Dove, M. C. (2009). The changing fate of oyster culture in New South Wales, Australia. Journal of Shellfish Research, 28, 803–811. 10.2983/035.028.0409

[ece34416-bib-0039] OEH (2016). Assessing estuary ecosystem health: Sampling, data analysis and reporting protocols Office of Environment and Heritage, Sydney. pp. 1–36, ISBN 978‐1‐76039‐344‐1

[ece34416-bib-0040] Perez‐Huerta, A. , & Cusack, M. (2009). Optimizing electron backscatter diffraction of carbonate biominerals‐resin type and carbon coating. Microscopy and Microanalysis, 15, 197–203. 10.1017/S1431927609090370 19460175

[ece34416-bib-0041] Perkins, A. K. , Santos, I. R. , Sadat‐Noori, M. , Gatland, J. R. , & Maher, D. T. (2015). Groundwater seepage as a driver of CO2 evasion in a coastal lake (Lake Ainsworth, NSW, Australia). Environmental Earth Sciences, 74, 779–792. 10.1007/s12665-015-4082-7

[ece34416-bib-0042] Port Stephens Council (2008). Tilligerry Creek Management Plan. (ed. ETEP Ltd ), pp. 1–92, ABN 61 089 482 888.

[ece34416-bib-0043] Ries, J. B. (2011). A physicochemical framework for interpreting the biological calcification response to CO_2_‐induced ocean acidification. Geochimica et Cosmochimica Acta, 75, 4053–4064. 10.1016/j.gca.2011.04.025

[ece34416-bib-0044] Ries, J. B. , Cohen, A. L. , & Mccorkle, D. C. (2009). Marine calcifiers exhibit mixed responses to CO2‐induced ocean acidification. Geology, 37, 1131–1134. 10.1130/G30210A.1

[ece34416-bib-0045] Sammut, J. , White, I. , & Melville, M. D. (1996). Acidification of an estuarine tributary in Eastern Australia due to drainage of acid sulfate soils. Marine and Freshwater Research, 47, 669–684. 10.1071/MF9960669

[ece34416-bib-0046] Scanes, E. , Parker, L. M. , O'Connor, W. A. , Stapp, L. S. , & Ross, P. M. (2017). Intertidal oysters reach their physiological limit in a future high‐CO_2_ world. The Journal of Experimental Biology, 220, 765–774. 10.1242/jeb.151365 28250175

[ece34416-bib-0047] Shirai, K. , Schöne, B. R. , Miyaji, T. , Radarmacher, P. , Krause, R. A. , & Tanabe, K. (2014). Assessment of the mechanism of elemental incorporation into bivalve shells (Arctica islandica) based on elemental distribution at the microstructural scale. Geochimica et Cosmochimica Acta, 126, 307–320. 10.1016/j.gca.2013.10.050

[ece34416-bib-0048] Skemer, P. , Katayama, I. , Jiang, Z. , & Karato, S.‐I. (2005). The misorientation index: Development of a new method for calculating the strength of lattice‐preferred orientation. Tectonophysics, 411, 157–167. 10.1016/j.tecto.2005.08.023

[ece34416-bib-0049] Smith, C. S. , & Heggie, D. T. (2003). Benthic Nutrient Fluxes in Wallis Lake. Geoscience Australia, Record 2003/22, ISBN: 0642467803.

[ece34416-bib-0050] Sonnenholzner, S. , & Boyd, C. E. (2000). Chemical and Physical Properties of Shrimp Pond Bottom Soils in Ecuador. Journal of the World Aquaculture Society, 31, 358–375.

[ece34416-bib-0051] Webb, J. R. , Santos, I. R. , Tait, D. R. , Sippo, J. Z. , Macdonald, B. C. T. , Robson, B. , & Maher, D. T. (2016). Divergent drivers of carbon dioxide and methane dynamics in an agricultural coastal floodplain: Post‐flood hydrological and biological drivers. Chemical Geology, 440, 313–325. 10.1016/j.chemgeo.2016.07.025

[ece34416-bib-0052] Wong, V. N. L. , Johnston, S. G. , Burton, E. D. , Bush, R. T. , Sullivan, L. A. , & Slavich, P. G. (2010). Seawater causes rapid trace metal mobilisation in coastal lowland acid sulfate soils: Implications of sea level rise for water quality. Geoderma, 160, 252–263. 10.1016/j.geoderma.2010.10.002

[ece34416-bib-0053] Yoshimura, T. , Tamenori, Y. , Suzuki, A. , Nakashima, R. , Iwasaki, N. , Hasegawa, H. , & Kawahata, H. (2013). Element profile and chemical environment of sulfur in a giant clam shell: Insights from μ‐XRF and X‐ray absorption near‐edge structure. Chemical Geology, 352, 170–175. 10.1016/j.chemgeo.2013.05.035

