# Supplementary material for: Coastal acidification impacts on shell mineral structure of bivalve mollusks
Source: Ecol Evol. 2018 Aug 14;8(17):8973–84. doi: 10.1002/ece3.4416 (PMC6157695; doi:10.1002/ece3.4416)
Supplement: Supplementary file 1 [file ECE3-8-8973-s001.docx]

**Supplementary Materials**

**Water parameters**

Data on water parameters are available from three separate sources. Data collected at each site at the same time as the oysters (Supp. Table 1) highlight how the sulphate soils floodplain affect the salinity, pH and analysed total alkalinity of the estuaries (Supp. Table 1). Low total alkalinity has previously been noted in Wallis Lake (Smith & Heggie, 2003) and here we can see the extremes of pH and total alkalinity in the Upper Wallamba site where oysters were growing despite the low pH (7.45) and low salinity (1472 ± 13 µmolkg^-1^) environments. In NSW extreme low pH has previously been reported as a direct result of sulphate soil run-off during heavy rainfall (Manning River, pH 5.47 – 8.12) (Dove & Sammut, 2007). We sampled water in triplicate at the time of oyster collection for each of three sites (Supp. Table 1). Temperature, salinity and pH were measured on site using a pH probe calibrated on the total pH scale (Dickson *et al.*, 2007). Total alkalinity was analysed later using standard semi – automated titration, combined with spectrometric analysis using bromocresol indicator (Dickson *et al.*, 2007, Fitzer *et al.*, 2015, Yao & Byrne, 1998).

**Supp. Table 1. Estuarine water parameters for each oyster sampling site. The salinity, temperature, pH and total alkalinity were measured in triplicate from the oyster sampling sites at the time of oyster collection and used to calculate the carbonate chemistry parameters using CO2SYS in the total pH scale (Riebesell *et al.*, 2010).**

| **Site** | | | **Salinity**  **PPT** | | **Temperature**  **°C** | | **pH** | **Total alkalinity**  **µmol kg^-1^** | | | **CO_3_^2-^**  **(µmol kg^-1^)** | | **ΩCa** | **ΩAr** | **pCO_2_**  **(µatm)** |
| --- | --- | --- | --- | --- | --- | --- | --- | --- | --- | --- | --- | --- | --- | --- | --- |
| **Wallis lake, Cockatoo Island ‘Control’** | | | **33.7** | | **20.4** | | **8.21** | **2199 ± 3** | | | **233.6** | | **5.64** | **3.66** | **242.2** |
| **Wallis lake, Upper Wallamba**  **‘Acidified site 1’** | | | **16.1** | | **20.0** | | **7.45** | **1472 ± 13** | | | **21.2** | | **0.58** | **0.35** | **1407.1** |
| **Port Stephens, Tilligerry Creek**  **‘Acidified site 2’** | | | **26.6/ 25.9** | | **17.8/ 18.1** | | **7.81/ 7.67** | **1897 ± 5** | | | **74.3/ 53.7** | | **1.87/ 1.36** | **1.18/ 0.86** | **656.7/ 962.5** |
|  |  |  | |  | |  | | |  |  | |  |  |  |  |

In addition, Port Stephens has been sampled extensively for water quality pH profiles to quantify the extent and duration of estuarine acidification (Dove & Sammut, 2013). It has been reported that Port Stephens has a median pH of 7.28, at temperature 22.86 (Port Stephens pH range of pH 4.31 - pH 8.44, n = 15,402 site water quality measurements) (Dove & Sammut, 2013)and that the duration of estuarine acidification at these levels are likely to cause long-term impacts on oysters (Dove & Sammut, 2013).

In austral summer 2016/2017 NSW Office of Environment and Heritage (OEH) measured pH, salinity, chlorophyll a, fDOM (a measure of tannins) and dissolved oxygen (Supp. Table 2) using an EXO 2 multiprobe calibrated according to manufacturer’s recommendations. These data are also presented in Figure 1 for Port Stephens and Figure 2 for Wallis Lake. In Figure 1, Port Stephens represents a ‘control’ estuarine site (e.g. latitude -32.705408, longitude 152.01838), and Tilligerry Creek represents the ‘acidified site 2’ of oyster collection (e.g. latitude -32.777738, longitude 151.954038). In Wallis lake, Wallamba Cove represents a similar acidified location in Upper Wallamba ‘acidified site 1’close to our oyster sample site (e.g. latitude -32.178308, longitude 152.485065) and Wallis Lake represents a ‘control’ estuarine site (e.g. latitude -32.320742, longitude 152.5064).

**Supp. Table 2. Water quality data collected from Port Stephens and Wallis Lake (OEH MER program), the data used for Figures 1 and 2 are presented below.**

| Estuary Name | Date (DD/MM/YYYY) | Probe pH | Probe Salinity (PPT) | Probe Chlorophyll a (ug/L) | Probe fDOM (RFU) | Probe fDOM (QSU) | Probe DO % |
| --- | --- | --- | --- | --- | --- | --- | --- |
| Port Stephens | 9/11/2016 | 8.07 | 35.72 | 1.10 | 0.58 | 2.38 | 97.27 |
| Port Stephens | 9/11/2016 | 8.08 | 35.73 | 1.31 | 0.72 | 2.81 | 97.78 |
| Port Stephens | 12/12/2016 | 8.02 | 35.83 | 0.75 | 1.03 | 3.77 | 95.43 |
| Port Stephens | 12/12/2016 | 7.94 | 36.29 | 1.21 | 2.94 | 9.58 | 96.46 |
| Port Stephens | 11/01/2017 | 8.02 | 35.77 | 1.56 | 1.17 | 4.19 | 94.78 |
| Port Stephens | 11/01/2017 | 7.93 | 36.16 | 1.86 | 2.59 | 8.50 | 96.91 |
| Port Stephens | 2/02/2017 | 7.92 | 35.85 | 0.97 | 1.28 | 4.54 | 92.12 |
| Port Stephens | 2/02/2017 | 7.84 | 36.24 | 2.88 | 2.62 | 8.61 | 92.51 |
| Port Stephens | 23/02/2017 | 8.04 | 35.86 | 1.28 | 0.88 | 3.29 | 97.62 |
| Port Stephens | 23/02/2017 | 7.99 | 36.18 | 2.07 | 1.83 | 6.18 | 95.04 |
| Port Stephens | 29/03/2017 | 7.91 | 30.40 | 4.04 | 8.43 | 26.30 | 99.88 |
| Port Stephens | 29/03/2017 | 7.96 | 32.53 | 1.61 | 4.68 | 14.86 | 92.69 |
| TILLIGERRY CREEK | 9/11/2016 | 7.57 | 30.80 | 9.69 | 17.52 | 53.97 | 95.37 |
| TILLIGERRY CREEK | 9/11/2016 | 7.70 | 32.51 | 4.64 | 12.37 | 38.28 | 93.02 |
| TILLIGERRY CREEK | 9/11/2016 | 7.82 | 33.75 | 4.42 | 6.95 | 21.79 | 94.42 |
| TILLIGERRY CREEK | 12/12/2016 | 7.44 | 33.57 | 8.41 | 21.15 | 65.04 | 74.26 |
| TILLIGERRY CREEK | 12/12/2016 | 7.58 | 34.17 | 6.32 | 18.72 | 57.64 | 83.31 |
| TILLIGERRY CREEK | 12/12/2016 | 7.73 | 34.92 | 4.24 | 13.27 | 41.03 | 89.08 |
| TILLIGERRY CREEK | 11/01/2017 | 7.50 | 34.77 | 10.30 | 20.06 | 61.71 | 75.68 |
| TILLIGERRY CREEK | 11/01/2017 | 7.57 | 35.12 | 9.18 | 18.07 | 55.64 | 78.28 |
| TILLIGERRY CREEK | 11/01/2017 | 7.70 | 35.07 | 5.10 | 12.11 | 37.44 | 86.84 |
| TILLIGERRY CREEK | 2/02/2017 | 7.34 | 36.25 | 12.94 | 23.60 | 72.48 | 69.41 |
| TILLIGERRY CREEK | 2/02/2017 | 7.47 | 36.60 | 8.31 | 21.38 | 65.74 | 76.16 |
| TILLIGERRY CREEK | 2/02/2017 | 7.65 | 30.47 | 5.54 | 12.62 | 39.07 | 86.84 |
| TILLIGERRY CREEK | 23/02/2017 | 7.83 | 37.29 | 7.80 | 15.84 | 48.86 | 89.52 |
| TILLIGERRY CREEK | 23/02/2017 | 7.90 | 37.32 | 6.01 | 14.69 | 45.37 | 94.48 |
| TILLIGERRY CREEK | 23/02/2017 | 7.87 | 37.04 | 4.54 | 10.68 | 33.16 | 92.69 |
| TILLIGERRY CREEK | 29/03/2017 | 7.41 | 25.04 | 11.39 | 29.02 | 88.99 | 86.00 |
| TILLIGERRY CREEK | 29/03/2017 | 7.62 | 27.64 | 6.46 | 22.81 | 70.09 | 88.28 |
| TILLIGERRY CREEK | 29/03/2017 | 7.69 | 28.78 | 4.36 | 17.77 | 54.73 | 90.56 |
| Wallamba Cove | 8/11/2016 | 7.92 | 35.16 | 2.93 | 3.40 | 10.99 | 84.57 |
| Wallamba Cove | 8/11/2016 | 7.86 | 35.17 | 1.11 | 3.41 | 11.00 | 87.30 |
| Wallamba Cove | 13/12/2016 | 7.90 | 35.40 | 5.82 | 3.79 | 12.16 | 96.62 |
| Wallamba Cove | 13/12/2016 | 7.91 | 35.54 | 3.96 | 3.53 | 11.38 | 99.12 |
| Wallamba Cove | 10/01/2017 | 7.89 | 35.38 | 6.31 | 4.47 | 14.23 | 86.92 |
| Wallamba Cove | 10/01/2017 | 7.87 | 35.09 | 3.53 | 4.01 | 12.85 | 90.32 |
| Wallamba Cove | 30/01/2017 | 7.85 | 35.47 | 9.83 | 4.12 | 13.18 | 106.31 |
| Wallamba Cove | 30/01/2017 | 7.84 | 35.63 | 3.27 | 3.69 | 11.87 | 100.13 |
| Wallamba Cove | 21/02/2017 | 7.89 | 35.47 | 8.93 | 4.51 | 14.35 | 105.85 |
| Wallamba Cove | 21/02/2017 | 7.84 | 35.53 | 5.21 | 4.84 | 15.36 | 99.46 |
| Wallamba Cove | 28/03/2017 | 7.47 | 19.20 | 6.96 | 41.28 | 126.34 | 59.63 |
| Wallamba Cove | 28/03/2017 | 7.62 | 22.32 | 6.23 | 35.81 | 109.68 | 67.19 |
| Wallis Lake | 8/11/2016 | 8.02 | 34.63 | 1.69 | 6.85 | 21.49 | 102.82 |
| Wallis Lake | 8/11/2016 | 8.16 | 35.21 | 0.85 | 3.02 | 9.83 | 106.54 |
| Wallis Lake | 8/11/2016 | 8.32 | 35.01 | 0.78 | 3.93 | 12.60 | 105.24 |
| Wallis Lake | 13/12/2016 | 8.11 | 36.77 | 3.92 | 8.58 | 26.76 | 118.60 |
| Wallis Lake | 14/12/2016 | 8.12 | 37.00 | 0.75 | 4.40 | 14.04 | 92.39 |
| Wallis Lake | 14/12/2016 | 8.34 | 37.38 | 3.82 | 5.80 | 18.28 | 89.33 |
| Wallis Lake | 14/12/2016 | 8.07 | 36.95 | 0.97 | 4.26 | 13.60 | 92.55 |
| Wallis Lake | 10/01/2017 | 8.03 | 32.77 | 3.48 | 9.21 | 28.66 | 108.11 |
| Wallis Lake | 10/01/2017 | 8.19 | 37.13 | 1.71 | 4.17 | 13.31 | 106.60 |
| Wallis Lake | 10/01/2017 | 8.29 | 38.21 | 2.83 | 5.49 | 17.35 | 104.92 |
| Wallis Lake | 10/01/2017 | 8.16 | 37.02 | 1.99 | 3.96 | 12.67 | 100.06 |
| Wallis Lake | 31/01/2017 | 7.93 | 35.50 | 2.76 | 8.52 | 26.57 | 108.55 |
| Wallis Lake | 1/02/2017 | 7.99 | 38.01 | 2.59 | 4.91 | 15.59 | 81.96 |
| Wallis Lake | 1/02/2017 | 8.07 | 39.66 | 1.88 | 6.75 | 21.17 | 88.57 |
| Wallis Lake | 1/02/2017 | 8.06 | 37.99 | 3.19 | 5.02 | 15.90 | 94.79 |
| Wallis Lake | 22/02/2017 | 7.94 | 35.97 | 1.68 | 8.15 | 25.44 | 78.85 |
| Wallis Lake | 22/02/2017 | 8.11 | 38.24 | 1.59 | 4.99 | 15.82 | 91.95 |
| Wallis Lake | 22/02/2017 | 8.16 | 39.38 | 1.64 | 6.68 | 20.95 | 100.50 |
| Wallis Lake | 22/02/2017 | 8.08 | 38.11 | 2.79 | 4.99 | 15.82 | 89.68 |
| Wallis Lake | 28/03/2017 | 8.00 | 25.33 | 11.45 | 21.55 | 66.26 | 119.68 |
| Wallis Lake | 28/03/2017 | 7.88 | 28.73 | 6.97 | 16.19 | 49.93 | 103.59 |
| Wallis Lake | 28/03/2017 | 7.92 | 30.72 | 4.00 | 13.92 | 43.03 | 101.70 |
| Wallis Lake | 28/03/2017 | 7.82 | 28.38 | 6.28 | 16.02 | 49.40 | 94.44 |


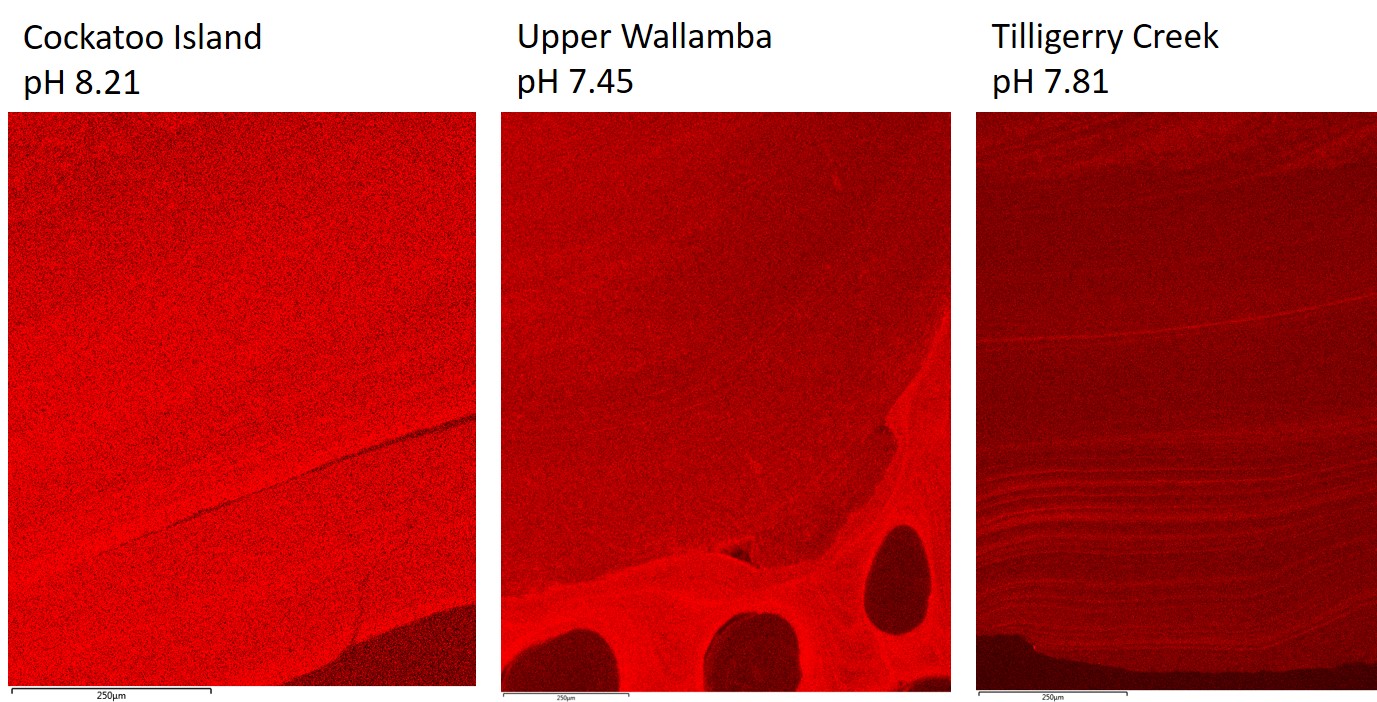


**Supp. Figure 1. Energy dispersive X-ray spectroscopy (EDS) total sulphur distribution maps indicate sulphur present in the shells in the range of <0.2 WT% for three representative shells from the ‘control’ Cockatoo Island site, and acidified Upper Wallamba ‘acidified site 1’ and Tilligerry Creek ‘acidified site 2’.**


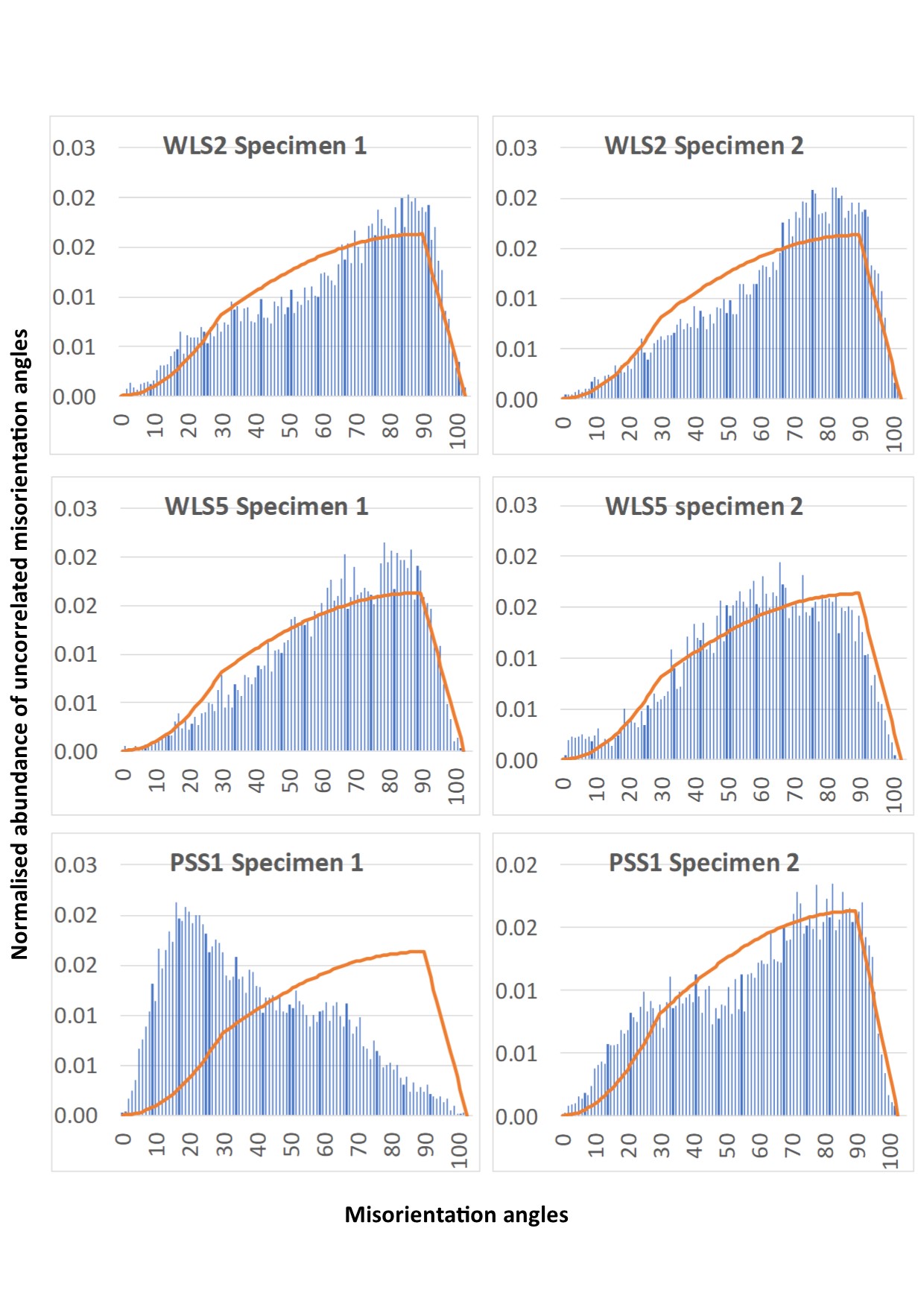


**Supp. Figure 2. Normalised abundance of uncorrellated misorientation angles against the misorientation angles. The blue bars are the measured data from 10,000 uncorellated misoreintations taken from each shell and the orange line is a theoretical normalised abundance of misorientation angles from a perfectly random distribution of hexagonal crystals.**

**References**

Dickson AG, Sabine CL, Christian JR (2007) *Guide to best practises for ocean CO_2_ measurements*.

Dove MC, Sammut J (2007) Impacts of estuarine acidfication on survival and growth of Sydney rock oysters, *Saccostrea glomerata* (GOULD 1850). Journal of Shellfish Research*,* **26**, 519-527.

Dove MC, Sammut J (2013) Acid Sulfate Soil Induced Acidification of Estuarine Areas Used for the Production of Sydney Rock Oysters, *Saccostrea glomerata*. Journal of Water Resource and Protection*,* **5**, 16.

Fitzer SC, Zhu W, Tanner KE, Kamenos NA, Phoenix VR, Cusack M (2015) Ocean acidification alters the material properties of *Mytilus edulis* shells. Journal of the Royal Society Interface*,* **12**, 20141227.

Riebesell U, Fabry VJ, Hansson L, Gattuso J-P (eds) (2010) *Guide to best practices for ocean acidification research and data reporting,* Luxembourg, Publications Office of European Union.

Smith CS, Heggie DT (2003) Benthic Nutrient Fluxes in Wallis Lake. Geoscience Australia*,* **Record 2003/22**, ISBN: 0642467803.

Yao WS, Byrne RH (1998) Simplified seawater alkalinity analysis: Use of linear array spectrometers. Deep-Sea Research Part I-Oceanographic Research Papers*,* **45**, 1383-1392.
